# Combinatorial actions of bacterial effectors revealed by exploiting genetic tools in yeast

**DOI:** 10.15252/msb.20167447

**Published:** 2017-01-01

**Authors:** Alan Huett

**Affiliations:** ^1^ School of Life SciencesD102 Medical SchoolQueens Medical CentreUniversity of NottinghamNottinghamUK

**Keywords:** Chromatin, Epigenetics, Genomics & Functional Genomics, Genetics, Gene Therapy & Genetic Disease, Microbiology, Virology & Host Pathogen Interaction

## Abstract

While yeast has been extensively used as a model system for analysing protein–protein and genetic interactions, in the context of bacterial pathogenesis, the use of yeast‐based tools has largely been limited to identifying interactions between pathogen effectors and host targets. In their recent work, Ensminger and colleagues (Urbanus *et al*, 2016) use the combinatorial power of yeast genetics to systematically screen all known *Legionella pneumophila* effector proteins for effector–effector interactions. They provide new insights into how bacterial effectors balance host cell perturbation and describe mechanisms used by “meta‐effectors” to directly modulate target effector activity.

*Legionella pneumophila* is a human pathogen associated with outbreaks of respiratory disease, usually caused by exposure to contaminated water aerosols from air conditioning units or industrial cooling towers. It is a common inhabitant of aqueous natural environments, where it has evolved an unusual lifestyle, often residing within amoebae. This intracellular niche has resulted in *L. pneumophila* possessing a formidable range of secreted effector proteins—over 300 have been described to date. These effector proteins are delivered directly to the host cytoplasm via a specialised secretion system, allowing the bacterium to escape phagocytic destruction and live within the amoebae after engulfment. These same effectors are responsible for the survival of the pathogen in human macrophages, and therefore, understanding effector function is particularly relevant. So far, most studies of *Legionella* effectors have been performed at the level of individual effector proteins or mutants. Thus, relatively little is known about effector–effector interactions, either direct or via modulating similar host pathways. Some examples of effector–effector inhibition have been discovered, but no comprehensive studies looking for synthetic effector–effector phenotypes have been performed.

Genetic screens in yeast have been widely applied and helped elucidate many of the fundamental processes of cell biology, genetics and metabolism. More recently, they have been exploited at vast scale to analyse synthetic lethal or suppressor phenotypes and comprehensively identify gene–gene interactions across the entire yeast genome (Costanzo *et al*, 2010; van Leeuwen *et al*, 2016). These synthetic genetic array (SGA) experiments demonstrate the power of combining robotics, yeast genetics and automated phenotyping to establish links between genes and biological processes in an unbiased manner. Bacterial effector function has also been explored in yeast and has identified novel effector proteins and elucidated their function in specific cellular pathways (Alto *et al*, 2006; Kramer *et al*, 2007; Slagowski *et al*, 2008). Indeed, a combination of yeast synthetic lethal genetics and bacterial effector expression has been used to rapidly place effectors within host pathways, by identifying yeast mutants hypersensitive to effector expression (Bosis *et al*, 2011). Importantly, these studies also illustrate the ability of many secreted bacterial effectors to retain function and correct protein folding when exogenously expressed in yeast, indicating that yeast is a good proxy for the diverse eukaryotic hosts of *Legionella*, and a suitable tool for high‐throughput approaches.

Urbanus *et al* (2016) extend this work to all pairwise combinations of 330 *L. pneumophila* type 4 secreted effectors, resulting in the comprehensive analysis of 108,000 potential interactions. Using techniques developed for SGA screens, they mated yeast expressing arrayed effector libraries to co‐express all possible pairs of effectors (Fig 1). Individually, many of these effectors cause a pronounced inhibition of yeast growth, making the screen well‐placed to identify suppressors of effector action.

**Figure 1:**
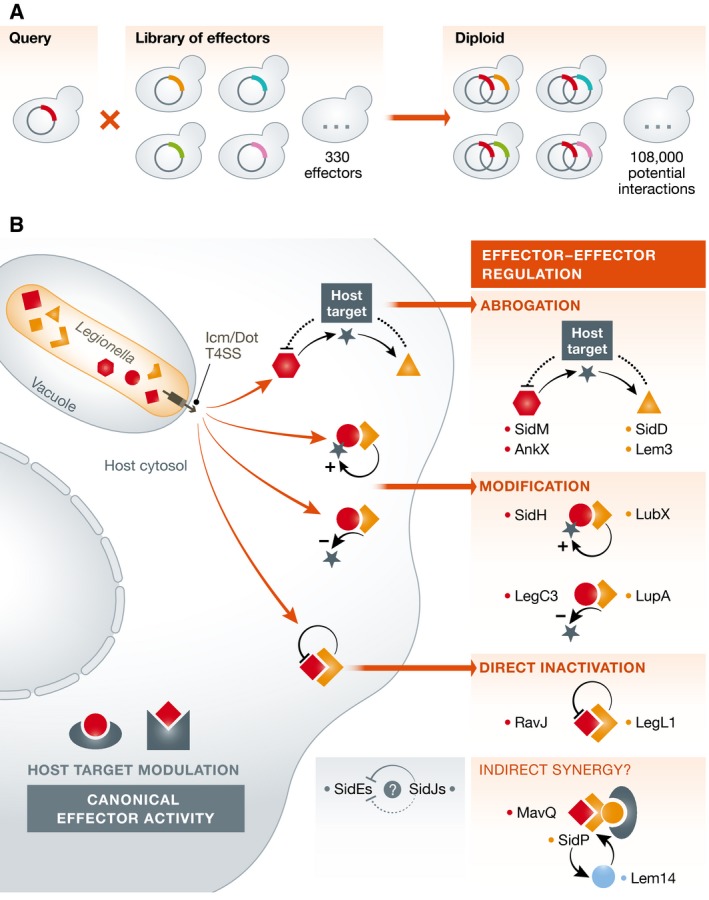
Finding effector–effector interactions in yeast (A) High‐throughput effector–effector suppression screen in yeast. Strains expressing an individual effector expressed from an inducible plasmid were mated with a library of 330 effectors, resulting in the comprehensive mapping of 108,000 pairwise effector–effector genetic interactions. (B) Effectors regulate one another using diverse mechanisms, including indirect interactions, that is by counteracting modification of a shared host target, or direct interactions involving either steric complex formation or direct modification of one effector by another.

This strategy was highly successful, recovering all six known antagonistic effector pairs, along with seventeen novel suppressor interactions. Interestingly, they also identified a synergistic interaction between SidP and Lem14—two effectors that do not inhibit growth when expressed individually and do not interact physically.

Further analyses using yeast two‐hybrid and other interaction assays demonstrated that nine effector–effector suppression phenotypes were mediated via direct, physical contact between effector proteins. These direct effector–effector interactions were termed meta‐effectors to distinguish them from effectors that act antagonistically via a shared target or pathway. In three cases, structural biology approaches, including X‐ray crystallography and homology modelling, identified protein function and key catalytic residues of both meta‐effectors and their cognate effectors. In one such case, that of meta‐effector LupA, the crystal structure revealed a deubiquitinase activity crucial for the inactivation of its partner effector LegC3. This is the first example of a deubiquitinating effector directly modulating another effector protein and raises the question whether other known bacterial deubiquitinating effectors also modify effectors in addition to host targets.

This study provides a fascinating insight into the landscape of secreted *L. pneumophila* effector proteins and their diverse roles in host cells. Several effectors have dual roles and modulate both host targets and the activity of other effectors. This represents a potential mechanism to finely tune the manipulation of the host by altering the relative levels of effector secretion, thus changing the balance of effector–effector versus effector–host interactions. Effector–effector balance could also change over time through differential effector half‐life, much as actin rearrangement is modulated during *Salmonella* infection (Kubori & Galán, 2003). The Urbanus *et al* (2016) study also elegantly illustrates the multidisciplinary power of integrating high‐throughput genetic and cell biology tools in molecular microbiology.
